# Pediatric primary ciliary dyskinesia with rare genetic variants: Synonymous *RSPH4A* and potential NFE2L2 modifier of *DNAH9* phenotype – 2-case report

**DOI:** 10.1097/MD.0000000000049153

**Published:** 2026-06-05

**Authors:** Cheng Guo, Yuyao Zhu, Hong Lu, Kai Liu

**Affiliations:** a Comprehensive Pediatrics, Kunming Children’s Hospital & Children’s Hospital of Kunming Medical University, Kunming City, Yunnan Province, China.

**Keywords:** NFE2L2 modifier effect, primary ciliary dyskinesia, *RSPH4A* synonymous mutation

## Abstract

**Rationale:**

Primary ciliary dyskinesia (PCD) is easily underdiagnosed in children without laterality defects. We report 2 pediatric cases of PCD, highlighting a candidate pathogenic synonymous *RSPH4A* variant and a possible modifier effect of NFE2L2 on *DNAH9*-related disease.

**Patient concerns:**

Case 1: a 5-year-old girl presented with chronic wet cough and recurrent pulmonary consolidations. Case 2: a 5-year-old boy presented with chronic wet cough, recurrent wheeze, and rhinosinusitis.

**Diagnoses:**

Case 1 had a normal immune work-up and underwent trio whole-exome sequencing (WES), which identified compound heterozygous *RSPH4A* variants (c.1391G>A and the synonymous c.1764G>T variant with a high predicted splicing impact), supporting a diagnosis of PCD. Case 2 had markedly elevated immunoglobulin E and an obstructive ventilatory defect; WES identified compound heterozygous *DNAH9* variants together with an NFE2L2 exon 2–5 deletion, consistent with PCD with enhanced type 2 inflammation.

**Interventions:**

Case 1 received antibiotic therapy. Case 2 was treated with antibiotics, bronchodilators, and inhaled corticosteroids.

**Outcomes:**

Case 1 improved after antibiotic therapy, and no bronchiectasis was detected during follow-up. Case 2 also improved clinically, although moderate airflow obstruction persisted.

**Lessons:**

These cases suggest that synonymous variants with strong splicing predictions in PCD genes may be pathogenic and that NFE2L2-related pathways may modify *DNAH9*-associated PCD severity. Early WES may facilitate diagnosis in children with chronic wet cough and recurrent infections, even without laterality defects.

## 1. Introduction

Primary ciliary dyskinesia (PCD) is a group of rare genetic disorders caused by structural or functional defects in motile cilia, with an estimated prevalence of approximately 1 in 7500 to 1 in 20,000.^[1,2]^ PCD patients may exhibit symptoms such as respiratory distress, recurrent rhinitis, pneumonia, and otitis media from infancy, with many developing bronchiectasis and impaired fertility in later life.^[1,3]^ Due to the high clinical heterogeneity of PCD and the absence of typical features such as laterality defects (e.g., situs inversus) in approximately half of patients, the current early diagnosis rate remains below 30%, with many patients not receiving a definitive diagnosis until adolescence.^[1,3–5]^ Timely identification of PCD is crucial for preventing irreversible lung damage.^[4,5]^ As of 2025, over 50 disease-causing genes have been identified, involving structures such as the ciliary outer and inner dynein arms and radial spokes.^[6,7]^ Whole-exome sequencing (WES) can simultaneously detect all known PCD-associated genes, and its diagnostic value in cases with atypical phenotypes is increasingly recognized.^[8–10]^ The 2 PCD cases reported in this study lacked typical laterality defects but were ultimately diagnosed through WES. This study aims to introduce 2 novel pathogenic gene combinations and explore their pathogenic mechanisms. Written informed consent for publication of the clinical details and genetic data was obtained from the parents of both patients.

## 2. Case report

### 2.1. Case 1

**Figure 1.**
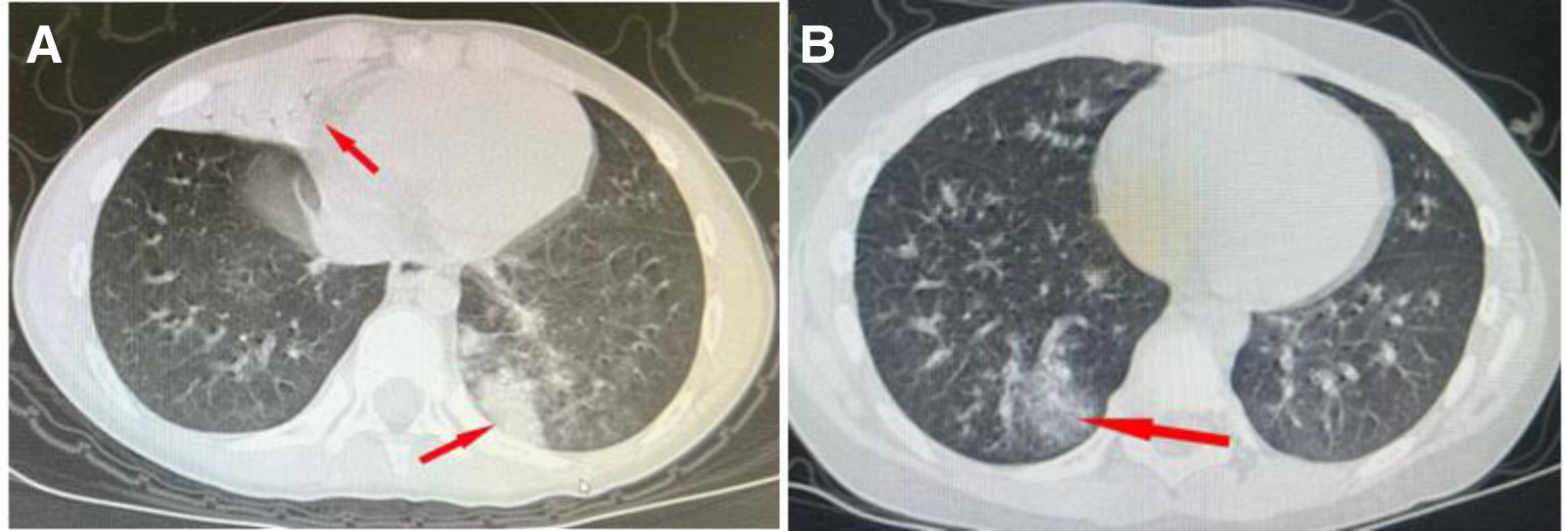
(A) Case 1: Chest CT of the 5-year-old girl showing recurrent right lower lobe inflammation and consolidation. (B) Case 2: Chest CT of the 5-year-old boy showing inflammatory changes with consolidation in both lungs. CT = computed tomography.

**Figure 2.**
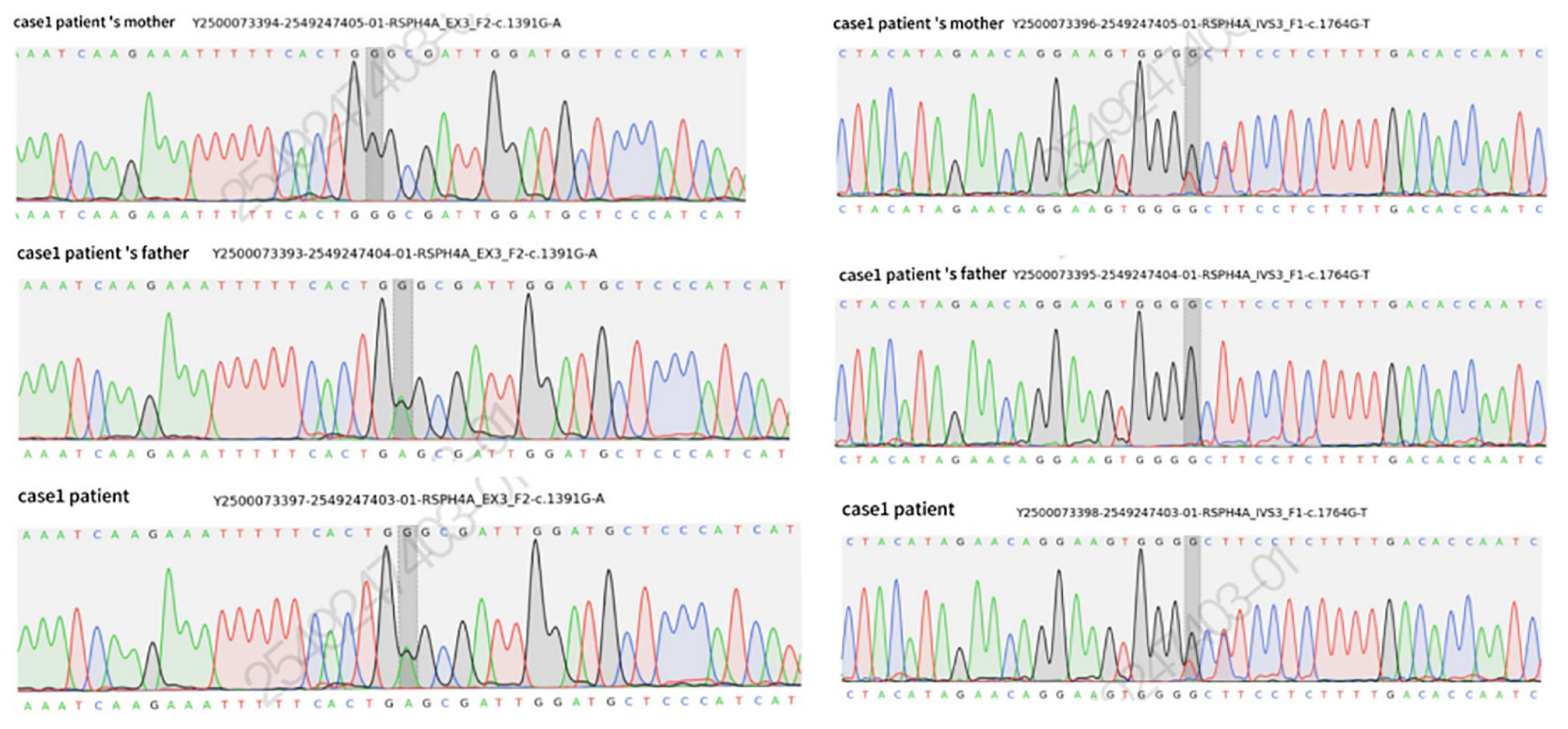
Case 1: First-generation sequencing validation in the 5-year-old girl.

A 5-year-old girl. Chief complaint: chronic wet cough for several months, particularly severe in the morning, with phlegm requiring repeated throat clearing for relief. History of present illness: since infancy, she has had recurrent chronic wet cough and persistent nasal congestion with purulent nasal discharge, with approximately 4 to 6 lower respiratory tract infections per year and multiple hospitalizations elsewhere for “pneumonia” or “bronchitis.” She developed respiratory distress on day 3 of life and was diagnosed with aspiration pneumonia, from which she recovered after a 5-day hospitalization. During the current episode, she presented with productive cough and intermittent exertional dyspnea. There was no history of otitis media, no consanguinity, and no family history of similar symptoms or situs abnormalities. Physical examination: scattered moist rales with occasional wheeze were heard over both lung fields, without cyanosis, digital clubbing, or cardiac abnormalities. Clinical assessment and imaging indicated situs solitus with no laterality defects. Laboratory tests: C-reactive protein was 52 mg/L at admission, whereas the leukocyte count and immunologic evaluation were within normal ranges. Imaging: chest computed tomography during this admission and prior episodes demonstrated recurrent pulmonary consolidations, predominantly involving the right lower lobe, with patchy opacities and interval resolution on follow-up imaging (Fig. 1A). Airway evaluation: bronchoscopy with bronchoalveolar lavage excluded endobronchial obstruction, structural airway anomalies, and specific pathogens. Given the disproportionately frequent and focal recurrent consolidations, trio WES was performed. Genetic findings: WES identified compound heterozygous variants in *RSPH4A* – c.1391G>A (p.Gly464Glu) and c.1764G>T (p.Gly588=) – each inherited from one parent (Table 1, Fig. 2). The missense variant c.1391G>A met PM2/PM3 (moderate evidence of pathogenicity [American College of Medical Genetics and Genomics (ACMG) criteria])/PP3 criteria (supporting computational evidence of pathogenicity [ACMG criterion]), whereas the synonymous variant c.1764G>T showed a high splicing-impact prediction (SpliceAI Δ = 0.72), suggesting aberrant mRNA splicing. Parental testing confirmed that the variants were in trans. Diagnosis and outcome: based on the typical airway phenotype and biallelic *RSPH4A* variants, a diagnosis of PCD was established. After 7 days of targeted antimicrobial therapy, the pneumonia resolved, and the patient was discharged. During structured PCD follow-up, no bronchiectasis has been detected to date.

**Table 1:** Case 1: *RSPH4A* compound heterozygous variant (WES result).

Chromosome	Gene	Variants and protein alteration	ACMG class	ACMG evidence	Causative	Diseases
chr6:116949261	*RSPH4A*	c.1391G>A (p.Gly464Glu)	Pathogenic	PM2, PM3, PP3	Compound heterozygous	Primary ciliary dyskinesia
chr6:116950831	*RSPH4A*	c.1764G>T (p.Gly588=)	Uncertain significance	PM2, PM3, PP3	Compound heterozygous	Primary ciliary dyskinesia

ACMG = American College of Medical Genetics and Genomics, PM2, PM3 = moderate evidence of pathogenicity (ACMG criteria), PP3 = supporting computational evidence of pathogenicity (ACMG criterion), WES = whole-exome sequencing.

### 2.2. Case 2

**Figure 3.**
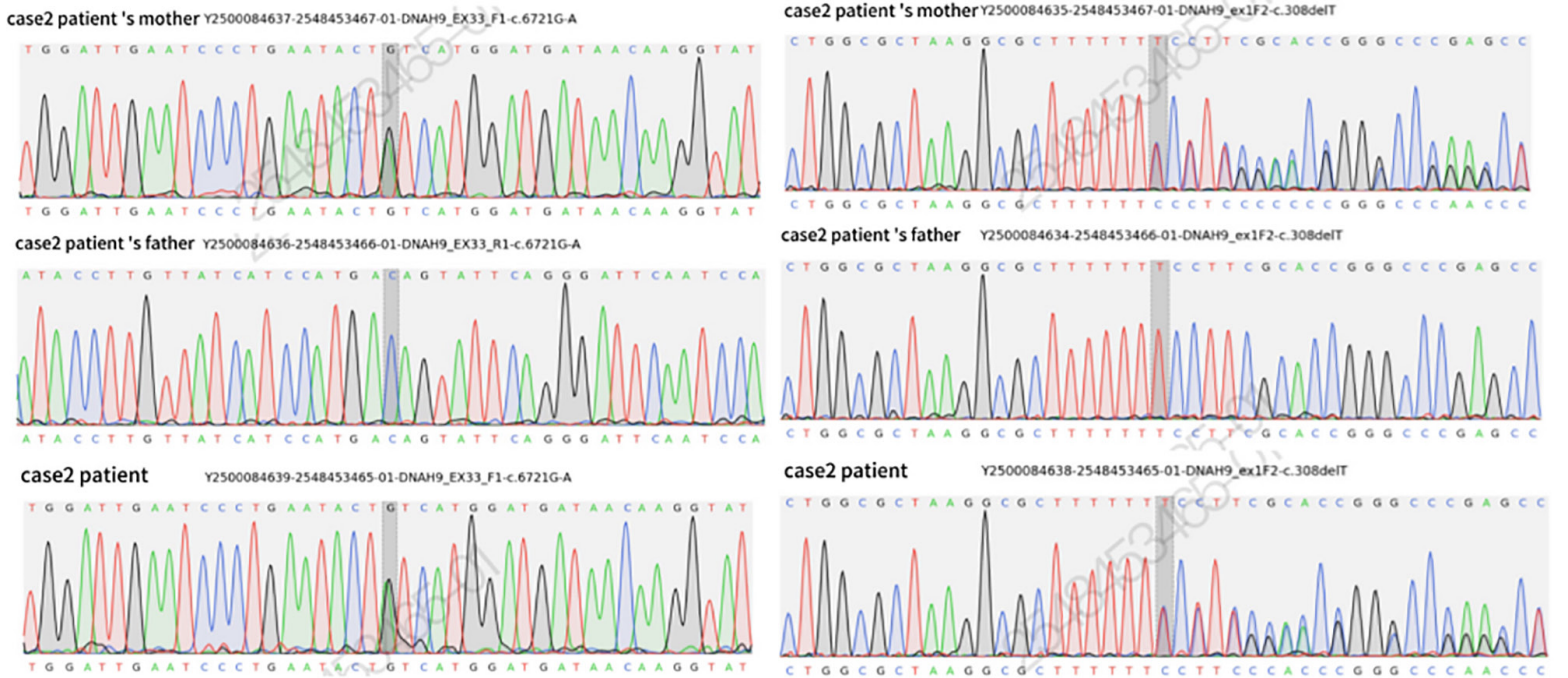
Case 2: First-generation sequencing validation in the 5-year-old boy.

A 5-year-old boy. Chief complaint: recurrent wheeze with chronic wet cough for more than 2 years, worsening over the preceding 2 weeks. History of present illness: since infancy, he has had a persistent wet cough, recurrent wheeze, and rhinosinusitis, with exacerbations triggered by seasonal changes or minor respiratory infections. Symptoms have been particularly prominent at night and in the early morning, and physical activity often provokes cough and shortness of breath. Caregivers reported copious phlegm that was difficult to expectorate, accompanied by recurrent postnasal drip and halitosis. The child has had multiple hospitalizations for “bronchitis” or “asthma.” Physical examination: bilateral expiratory wheeze, congested nasal mucosa with postnasal drip, and normal cardiac auscultation were noted, with no laterality defects identified clinically. Laboratory and functional evaluation: immunoglobulin E (IgE) was markedly elevated at 5607 IU/L. Pulmonary function testing demonstrated a moderate-to-severe obstructive ventilatory defect. Fractional exhaled nitric oxide was 51 ppb, and the bronchial provocation test was positive. Imaging: chest computed tomography demonstrated right lower lobe inflammatory changes together with sinus mucosal thickening (Fig. 1B), and nasopharyngoscopy confirmed sinusitis. Routine immunologic evaluation and cystic fibrosis screening were unremarkable. After stabilization of the acute episode, trio WES was performed. Genetic findings: WES identified compound heterozygous *DNAH9* variants – c.308delT (p.Phe103Serfs*31; pathogenic, PVS1 [very strong evidence of pathogenicity (ACMG criterion)] + PM2) and c.6721G>A (p.Val2241Ile; variant of uncertain significance, PM2 + PP3) – together with a heterozygous NFE2L2 exon 2–5 deletion (Table 2, Fig. 3). The *DNAH9* variants were confirmed in trans, whereas parental testing did not identify the NFE2L2 deletion, supporting a de novo event. Diagnosis and outcome: the combination of chronic wet cough, recurrent wheeze, rhinosinusitis, markedly elevated IgE, an obstructive ventilatory defect, and the above genetic findings supported a diagnosis of PCD with enhanced type 2 airway inflammation. The patient improved after treatment with antibiotics, bronchodilators, and inhaled corticosteroids; however, moderate airflow obstruction persisted during follow-up, indicating the need for long-term management.

**Table 2:** Case 2: *DNAH9* compound heterozygous variant and NFE2L2 deletion (WES results).

Chromosome	Gene	Variants and protein alteration	ACMG class	ACMG evidence	Causative	Diseases
chr17:11502117	*DNAH9*	c.308delT (p.Phe103Serfs*31)	Pathogenic	PVS1, PM2	Compound heterozygous	Primary ciliary dyskinesia
chr17:11656260	*DNAH9*	c.6721G>A (p.Val2241Ile)	Uncertain significance	PM2, PP3	Compound heterozygous	Primary ciliary dyskinesia
chr2:178095514-178098999	*NFE2L2*	EX2_EX5Del	Uncertain significance	PM2	Compound heterozygous	Immunodeficiency, developmental delay, hypohomocysteinemia

ACMG = American College of Medical Genetics and Genomics, PM2, PM3 = moderate evidence of pathogenicity (ACMG criteria), PVS1 = very strong evidence of pathogenicity (ACMG criterion), WES = whole-exome sequencing.

## 3. Discussion

This study reports the first case of PCD in a patient carrying a synonymous variant (c.1764G>T) in the *RSPH4A* gene and suggests that this variant may contribute to disease through abnormal splicing, thereby expanding the spectrum of potentially pathogenic variants in this gene.^[11–13]^ *RSPH4A* encodes the radial spoke head protein of cilia, a key molecule for maintaining the structural stability of 9 + 2 motile cilia.^[12,13]^ Previous studies have primarily reported missense or splice-site mutations as pathogenic variants, with no reports of pathogenic synonymous variants.^[13]^ Although the variant in this case does not alter the amino acid sequence, SpliceAI predicts a splicing score of Δ = 0.72, suggesting potential disruption of normal mRNA splicing sites.^[14,15]^ Previous studies have shown that a non-trivial fraction of pathogenic variants can be synonymous single-nucleotide variants (sSNVs), acting via splicing disruption, mRNA degradation, or altered translation efficiency.^[15–17]^ Considering the patient’s typical phenotype, compound heterozygous variant, and clear family segregation, this study provides clinical evidence for synonymous variant-mediated PCD pathogenesis, in keeping with current variant interpretation frameworks.^[18]^ The patient had no laterality defects, consistent with the molecular characteristics that *RSPH4A* affects 9 + 2 motile cilia but not the embryonic 9 + 0 nodal cilia responsible for laterality determination.^[19–21]^ Currently, there is a lack of functional validation data such as ultrastructural analysis or ciliary motility studies; further cDNA splicing analysis is planned to clarify transcriptional impact.^[14,15]^ In this context, the c.1764G>T change should be regarded as a strong candidate variant rather than definitively proven pathogenic, and functional studies will be essential to confirm its effect. This finding emphasizes that the pathogenic potential of sSNVs should not be overlooked in clinical practice, especially in patients with refractory PCD who test negative for known pathogenic variants.^[16,17]^ In previously reported *RSPH4A*-related PCD, patients commonly present with chronic wet cough and upper-airway disease, whereas laterality defects are usually absent, consistent with the biology that *RSPH4A* affects 9 + 2 motile cilia rather than 9 + 0 nodal cilia.^[11–13]^ Case 1 mirrors this pattern as a girl with chronic wet cough, recurrent pulmonary consolidations, a normal immune work-up, and compound heterozygous *RSPH4A* variants, without laterality defects, further supporting close scrutiny of high-confidence sSNVs during WES/whole genome sequencing interpretation.^[14,15]^

The genetic background of the second patient included compound heterozygous *DNAH9* variants together with a heterozygous NFE2L2 deletion, suggesting that ciliary structural defects and inflammatory regulation disorders may synergistically exacerbate the clinical phenotype of PCD.^[22]^ *DNAH9* encodes the heavy chain of the ciliary outer dynein arm, which contributes to distal ciliary rhythmic beating.^[22,23]^ Previous *DNAH9* mutations have mostly been associated with relatively mild PCD phenotypes, with few reports of significant lung function impairment and bronchiectasis before age 5 years.^[24]^ The patient carries a frameshift mutation predicted to cause protein truncation (PVS1) and a C-terminal missense variant (PM2 + PP3), which could theoretically retain partial function; however, in the context of concurrent NFE2L2 heterozygous deletion (exons 2–5), the respiratory phenotype was markedly aggravated.^[18,24]^ NFE2L2 encodes NRF2, a core transcriptional regulator of antioxidant and anti-inflammatory responses. NRF2 deficiency in animal models leads to Th2-biased immunity, markedly elevated IgE, and enhanced airway hyperresponsiveness, consistent with this patient’s presentation.^[25,26]^ We speculate that disease severity is not fully explained by *DNAH9* variants alone; rather, reduced NRF2 signaling increases airway susceptibility and sustains inflammation, amplifying damage secondary to impaired mucociliary clearance – forming a putative synergistic pathogenic pattern of “ciliary defect + inflammatory modification.”^[26]^ However, this model remains hypothetical: direct functional evidence for NFE2L2 as a PCD modifier is lacking, and its role requires confirmation in larger, systematically characterized PCD cohorts in conjunction with functional studies.^[27]^ By contrast, published *DNAH9* cohorts usually show milder respiratory phenotypes with later diagnoses.^[22,23]^ In case 2, a boy with chronic wet cough, recurrent wheeze, rhinosinusitis, markedly elevated IgE, an obstructive ventilatory defect, compound heterozygous *DNAH9* variants, and an NFE2L2 exon 2–5 deletion showed a more severe phenotype than is usually reported for *DNAH9*-related disease, aligning with experimental evidence that NRF2 deficiency drives Th2-biased airway inflammation and hyperresponsiveness.^[25,26]^ This divergence from the typical *DNAH9* course supports a “ciliary defect + inflammatory modification” model and reinforces guideline-endorsed, genetics-guided diagnostic pathways for atypical or non-situs PCD.^[28]^

Based on these 2 cases, we cautiously propose that, beyond classic ciliary structural defects, nontraditional genetic factors (e.g., synonymous variants and inflammation-regulating genes) can influence the clinical spectrum and severity of PCD.^[26]^ This observation supports reframing PCD from a “monogenic disorder” to a “heterogeneous disease driven by a complex genetic background.” In the current context of widespread WES deployment for atypical phenotypes, identifying potential modifying factors has practical value for improving early detection and personalized management.^[28,29]^ Clinically, 2 strategies are underscored: prioritize sSNVs with strong splicing-impact predictions, integrating family data and phenotype for pathogenicity assessment; and in severe phenotypes (e.g., very high IgE or refractory airway hyperresponsiveness), consider targeted screening of inflammation-regulatory genes to explore synergistic mechanisms.^[28]^ Future studies should expand sample sizes and incorporate functional validation to assess whether inflammatory regulatory genes, including *NFE2L2*, broadly modulate PCD phenotypes, advancing precise subtyping and risk stratification.^[30–33]^ These observations should therefore be regarded as hypothesis-generating and interpreted cautiously in light of the small sample size and absence of direct functional assays.

## 4. Conclusion

This study reports 2 cases of atypical PCD in children, one carrying an *RSPH4A* synonymous variant and the other carrying *DNAH9* compound heterozygous variants together with an NFE2L2 exon 2–5 deletion, suggesting that single-nucleotide variant–mediated splicing abnormalities and dysregulated inflammation may significantly impact the molecular diagnosis and phenotypic expression of PCD. These findings further suggest that NFE2L2-related pathways may modify the severity of *DNAH9*-associated PCD. In suspected cases lacking typical clinical features, early application of WES may facilitate molecular diagnosis and may provide clues to potential phenotypic modifiers. Overall, these cases support the consideration of more precise genetic assessment strategies in the clinical management of PCD and may further inform the understanding of phenotypic heterogeneity.

## Author contributions

**Conceptualization:** Yuyao Zhu, Hong Lu.

**Writing – original draft:** Cheng Guo.

**Writing – review & editing:** Kai Liu.
